# Recent advances in understanding inherited deficiencies in immunity to infections

**DOI:** 10.12688/f1000research.22036.1

**Published:** 2020-04-07

**Authors:** Gregory M. Constantine, Michail S. Lionakis

**Affiliations:** 1 Fungal Pathogenesis Section, Laboratory of Clinical Immunology and Microbiology, National Institute of Allergy and Infectious Diseases, National Institutes of Health, Bethesda, Maryland, 20814, USA

**Keywords:** immunodeficiency, inherited, monogenic, infection, immunology, infectious disease

## Abstract

The immune system is central to our interactions with the world in which we live and importantly dictates our response to potential allergens, toxins, and pathogens to which we are constantly exposed. Understanding the mechanisms that underlie protective host immune responses against microbial pathogens is vital for the development of improved treatment and vaccination strategies against infections. To that end, inherited immunodeficiencies that manifest with susceptibility to bacterial, viral, and/or fungal infections have provided fundamental insights into the indispensable contribution of key immune pathways in host defense against various pathogens. In this mini-review, we summarize the findings from a series of recent publications in which inherited immunodeficiencies have helped illuminate the interplay of human immunity and resistance to infection.

## Introduction

Insights through the observation and study of primary immunodeficiency disorders (PIDs) provide a unique *in vivo* lens through which our understanding of human immunology can be advanced. Herein, we discuss recent discoveries that have furthered our collective understanding of the host immune response in protection from the constant environmental threats posed by bacteria, viruses, and fungi. We focus on interleukin (IL)-6 receptor-alpha (IL-6Rα) deficiency and dominant activating Rac family small GTPase 2( *RAC2*) mutations leading to bacterial infection susceptibility, EROS deficiency and its impact on innate immunity, interferon (IFN) regulatory factor 4 (IRF4) haploinsufficiency causing susceptibility to Whipple’s disease (WD), defects in IL-12 receptor subunit beta 2 (IL-12RB2), IL-23 receptor (IL-23R), RAR-related orphan receptor C (RORC), Janus kinase 1 (JAK1), and tyrosine kinase 2 (TYK2) contributing to mycobacterial disease, deficiency in IFNα receptor-1 (IFNAR1), melanoma differentiation associated protein-5 (MDA5), IRF9, or IL-18 binding protein (IL18-BP) and gain-of-function (GOF) mutations in NLR family pyrin domain containing 1 ( *NLRP1*) leading to viral infection susceptibility, and fungal infection susceptibility in the setting of caspase recruitment domain-containing protein 9 (CARD9) deficiency, zinc finger protein 341 (ZNF341) deficiency, and c-Jun N-terminal kinase 1 (JNK1) haploinsufficiency.

## Recent insights gained from primary immunodeficiency disorders manifesting with bacterial infection susceptibility

### IL-6 signaling: a critical role in control of bacteria

Prompt recognition of bacterial pathogens is paramount in the defense against infection, and, among the mechanisms that initiate the immune cascade, IL-6 plays a central role. As a pleiotropic pro-inflammatory cytokine, IL-6 leads to the initiation of the acute-phase response by driving the recruitment of innate immune cells and pathogen phagocytosis while also promoting adaptive immune responses through mediating B cell survival and maturation, T cell proliferation, and the lineage commitment of T follicular, T _H_17, and T _H_22 helper cells ^1– 3^. Classical cell-membrane signaling of IL-6 is mediated through a hexameric complex composed of two IL-6 molecules, two IL-6Rα receptors, and two transmembrane glycoprotein 130 (gp130) proteins through which signal transduction is propagated via JAK and signal transducer and activator of transcription (STAT) interactions ^4– 6^. Iatrogenic, acquired, and inherited defects have provided evidence of the consequences of impaired IL-6 signaling through their impact on infection susceptibility. Severe staphylococcal infections have been described in patients treated with the anti-IL-6R monoclonal antibody tocilizumab and in spontaneously acquired autoantibodies against circulating IL-6 ^7– 9^. Furthermore, inherited homozygous mutations in *IL6ST,* encoding the gp130 co-receptor, predispose to recurrent severe staphylococcal and streptococcal infections ^10^ as well as elevated serum IgE and dental and cranio-skeletal manifestations reminiscent of hyper-IgE syndrome (HIES) caused by dominant-negative mutations in *STAT3* ^11, 12^. It was not until recently that inherited deficiency in IL-6Rα was identified. Specifically, Spencer *et al.* reported two patients with severe recurrent infections with defective acute-phase response, elevated IgE, atopic dermatitis, and peripheral eosinophilia caused by pathogenic variants in *IL6R* ^13^. An additional four patients from two unrelated families with *IL6R* mutations were also reported sharing a similar clinical phenotype with severe bacterial infectious complications caused by *Streptococcus pneumoniae*, *Staphylococcus aureus*, *Haemophilus influenzae*, and *Neisseria meningiditis* ^14^ ( Table 1). Mechanistically, shared among all of these IL-6 pathway functional defects is absent or delayed production of acute-phase reactants including C-reactive protein, which plays a crucial role in the protection against bacterial pathogens including Streptococci *, Klebsiella pneumoniae*, and Neisseria meningitidis through opsonization and phagocytosis by macrophages and neutrophils ^1, 15– 17^.

**Table 1.** Recently described primary immunodeficiencies associated with increased risk of infection

Gene/ protein	Variant(s)	Inheritance model	Clinical manifestations	Laboratory findings	Associated pathogens
**Host response to bacteria**					
*IL6R*/IL- 6RA	G183Efs*7;I279N	AR, LOF	Atopic dermatitis, respiratory infections, skin abscesses, recurrent skin infections	Variably low IgG, IgA ↑ IgE ↓ CRP	*Streptococcus* *pneumoniae*, *Staphylococcus aureus*, *Haemophilus influenzae*, *Neisseria meningitidis*
*IRF4* /IRF4	R98W	AD, haploinsufficiency	Whipple’s disease, chronic *Tropheryma whipplei* carriage	NR	*T. whipplei*
*RAC2* /RAC2	E62K; P34H	AD, GOF	Recurrent sinopulmonary infections, bronchiectasis, cellulitis, lymphadenitis, B cell lymphoma, littoral cell angioma	Neutropenia, lymphopenia, variable hypogammaglobulinemia, increased DHR	*N. meningitidis*, varicella zoster, HPV, herpes simplex
*CYBC1*/ EROS	c.127G>A; Y2X	AR, LOF	Short stature, skin/soft tissue infections, lymphadenitis, recurrent sinopulmonary infections, granulomatous inflammation, AIHA, HLH, CGD, IBD	Impaired DHR, lymphopenia	BCG, *Burkholderia* *cepacia, Legionella,* *Candida albicans, * *Clostridium difficile, * *Mycobacterium* *tuberculosis*
**Host response to mycobacteria**					
*IL12RB2*/ IL-12Rβ1	Q138X	AR, LOF	Lymphadenitis, pulmonary tuberculosis	Increased naïve CD4 ^+^ and CD8 ^+^ T cells	BCG, *M. tuberculosis*
*IL23R*/IL- 23R	C115Y	AR, LOF	Lymphadenitis, disseminated BCG infection	Increased naïve CD4 ^+^ and CD8 ^+^ T cells	BCG
*TYK2/* TYK2	P1104A	AR, LOF	Primary tuberculosis	Increased naïve CD4 ^+^ and CD8 ^+^ T cells, reduced effector memory T cells	*M. tuberculosis,* NTM
*RORC* /RORγ/ RORγt	Q308X; Q420X	AR, LOF	Thymic hypoplasia, CMC, disseminated BCG infection, tuberculosis, eczematous dermatitis, recurrent skin infections	CD4 ^+^ and CD8 ^+^T cell lymphopenia ↓IFN-γ production ↓ IL-17A/F, IL-21, IL-22 production	BCG, *M. tuberculosis,* *C. albicans*
*JAK1*/ JAK1	P733L; P832S	AR, LOF	Recurrent sinopulmonary infections, osteomyelitis, developmental delay, urothelial carcinoma, skin infections, flat warts, crusted scabies	Reduced naive CD4 ^+^ and CD8 ^+^ T cells, Impaired PHA response Impaired IFN-γ, IFN-α, IL- 2, IL-4, IL-10, and IL-27 responses ↓IFN-γ production ↑ IL-6 production	NTM, *Sarcoptes scabiei*, HPV
**Host response to viruses**					
*IFNAR1*/ IFNAR1	V225fs; W261X, V225_P23 2del	AR, LOF	Disseminated vaccine- strain measles, yellow fever vaccine-associated viscerotropic disease	Impaired type I IFN responses	Vaccine-strain measles, vaccine-strain yellow fever
*IFIH1*/ MDA5	K365E	AR, LOF	Recurrent viral respiratory infections	Reduced IFN-β, IFN-λ transcription	HRV, influenza B, influenza A, coronavirus, RSV, parainfluenza
*IRF9*/IRF9	E166Lfs*80, D331N	AR, LOF	Recurrent viral infections, severe influenza infection	Variable mild lymphopenia, variable hypogammaglobulinemia, impaired type I IFN responses	Influenza A
*IL18BP*/IL- 18 BP	c.508-19_528del	AR, LOF	HAV-induced acute liver failure	↑ IL-18	HAV
*NLRP1*/ NLRP1	T755N	AR, GOF	Recurrent respiratory papillomatosis, palmar and plantar warts	↑ IL-18 ↑TNF-α	HPV
*TLR3*/TLR3	P554S; P680L	AD, LOF	Acute respiratory distress syndrome	↓IFN-β and IFN-λ from fibroblasts	Influenza A, RSV
**Host response to fungi**					
*CARD9*/ CARD9	NA	AR, LOF	Mucocutaneous and CNS fungal infections	↓ CNS neutrophil recruitment due to impaired IL-1β/CXCL1 microglial production ↓ neutrophil killing against unopsonized *Candida* yeasts Impaired IL-17 responses	*C. albicans, Aspergillus* species, agents of dermatophytosis and phaeohyphomycosis
*ZNF341*/ ZNF341	R302X; K355fs; Y542X; Q195X	AR, LOF	CMC, staphylococcal skin infections, respiratory infections, bronchiectasis, severe atopic dermatitis, food and environmental allergy, connective tissue abnormalities	Low NK Cells Low memory B cells Low central memory CD4 ^+^ and CD8 ^+^ T cells Low IgM, IgA ↑ IgE	*S. aureus, C. albicans,* *Candida glabrata*
*MAPK8*/ JNK1	c.311+1G>A	AD, haploinsufficiency	Systemic connective tissue disorder, CMC, recurrent bacterial skin infections, urinary tract infections	Impaired IL-17A/F responses	*C. albicans,* Staphylococcal species

AD, autosomal dominant; AIHA, autoimmune hemolytic anemia; AR, autosomal recessive; BCG, Bacillus Calmette-Guérin; CARD9, caspase recruitment domain-containing protein 9; CGD, chronic granulomatous disease; CMC, chronic mucocutaneus candidiasis; CNS, central nervous system; CRP, C-reactive protein; DHR, dihydrorhodamine test; GOF, gain-of-function; HAV, Hepatitis A virus; HLH, hemophagocytic lymphohistiocytosis; HPV, human papilloma virus; HRV, human rhinovirus; IBD, inflammatory bowel disease; IFN, interferon; IFNAR1, interferon alpha receptor-1; Ig, immunoglobulin; IL, interleukin; IL-6RA, interleukin-6 receptor-alpha; IL-12Rβ1, interleukin-12 receptor subunit beta 1; IL-18 BP, interleukin-18 binding protein; IL-23R, interleukin-23 receptor; IRF, interferon regulatory factor; JAK1, Janus kinase 1; JNK1, c-Jun N-terminal kinase 1; LOF, loss-of-function; MDA5, melanoma differentiation associated protein-5; NA, not applicable; NK, natural killer; NLRP1, NLR family pyrin domain containing 1; NR, not reported; NTM, non-tuberculous mycobacteria; PHA, phytohemagglutinin; RAC2, Rac family small GTPase 2; ROR, RAR-related orphan receptor; RSV, respiratory syncytial virus; TNF, tumor necrosis factor; TLR3, Toll-like receptor-3; ZNF341, zinc finger protein 341.

As the IL-6 signaling pathway has been implicated in autoimmune and inflammatory diseases such as rheumatoid arthritis, giant cell arteritis, Castleman’s disease, and cytokine release syndrome in the setting of CAR T cell therapy for malignancies, anti-IL-6R therapies have become increasingly more common ^18^. As such, surveillance for bacterial infectious complications is warranted in the setting of anti-IL-6 biological therapies.

### Whipple’s disease: 111 years later

Described initially by George Hoyt Whipple ^19^, WD is a chronic infection manifested by malabsorptive diarrhea, weight loss, and arthritis caused by the Gram-positive bacillus *Tropheryma whipplei* ^20^. Despite *T. whipplei* being ubiquitous in the soil and water, WD is rare, with an estimated incidence of 1–3 cases per 1 million people ^21^. Interestingly, asymptomatic carriage is relatively frequent, ranging from ~4–31% depending on the geographical area ^22, 23^. The vast majority of infected individuals suffer from self-limited illness, suggesting that an impaired immunological response may play a role in those with chronic or invasive forms of the disease ^24– 27^.

**Figure 1.**
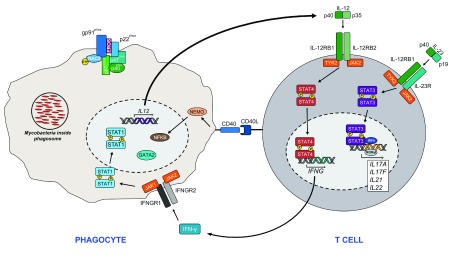
The IFN-γ/IL-12, IL-23, IL-17 axis and NADPH complex in host defense against pathogens Mycobacteria are recognized and phagocytosed, leading to IL-12 (p40/p35) secretion by macrophages. Binding of IL-12 to its receptor on the surface of T _H_1 (or NK) cells activates the downstream JAK2 and TYK2 signaling cascade, resulting in STAT4 phosphorylation and dimerization. STAT4 homodimers translocate to the nucleus and induce the transcription of IFN-γ. Phosphorylated STAT1 homodimers translocate to the nucleus, resulting in the transcription of host defense genes. Similarly, IL-23 comprising the p40 and p19 subunits binds to the IL-23 receptor, leading to JAK2/TYK2 activation and STAT3 phosphorylation, dimerization, and nuclear translocation, where it associates with IRF4 and RORγt in the transcription of *IL17*- *, IL21*- *,* and *IL22*-related genes. The membrane-bound gp91 ^phox^ and p22 ^phox^ heterodimer (cytochrome b558) is stabilized by the transmembrane chaperone protein EROS. The remaining cytosolic components, p67 ^phox^, p47 ^phox^, and p40 ^phox^, along with RAC2-GTP, associate to form the activated NADPH complex. Secondary signal from CD40/CD40L signaling activates the NF-κB pathway. GATA2, GATA binding factor 2; gp, glycoprotein; IFN, interferon; IFNG, interferon gamma; IFNGR, interferon gamma receptor; IL, interleukin; IL-12RB, IL-12 receptor subunit beta; IRF4, interferon regulatory factor 4; JAK2, Janus kinase 2; NAPDH, nicotinamide adenine dinucleotide; NEMO, nuclear factor-κB essential modulator; NF-κB, nuclear factor-κB; NK, natural killer; RAC2, Rac family small GTPase 2;RORγt, RAR-related orphan receptor γt; STAT, signal transducer and activator of transcription; T _H_1, T helper type 1; TYK2, tyrosine kinase 2. This figure is adapted and amended with permissions, from Figure 3 in Abers, A, Lionakis, M. Chronic mucocutaneous candidiasis and invasive fungal infection susceptibility. In: Sullivan, K.E, Stiehm, E.R *Stiehm's Immune Deficiencies-Inborn Errors of Immunit*. p1–44. 2nd ed. Copyright Elsevier Science & Technology. 2020 ^37^.

Through the investigation of four related patients with WD, Guérin *et al.* reported a rare loss-of-function (LOF) mutation in *IRF4* causing haploinsufficiency ^28^. It is important to note that a total of 12 individuals were found to be heterozygous for the p.R98W variant in IRF4 ( Table 1), including the four WD patients, five chronic *T. whipplei* carriers, two non-carriers, and one relative who was not tested for *T. whipplei,* thereby indicating incomplete penetrance. IRF4 is an important transcription factor for the development of immune cells as well as T _H_2, T _H_9, and T _H_17 responses ( Figure 1); however, its role in the control of bacterial infection has been less clear ^29, 30^. *In vitro* studies of patient cells demonstrated that the mutant IRF4 allele failed to bind DNA or induce transcription. Additional transcriptional analysis of *T. whipplei*-infected peripheral blood mononuclear cells (PBMCs) did not detect specific pathway enrichment; however, *Mycobacterium bovis*–Bacillus Calmette-Guérin (BCG)-infected PBMCs demonstrated downregulation of STAT1 and IFN-γ-associated pathways. These results suggest that IRF4 haploinsufficiency may predispose to *T. whipplei* via impaired T _H_1 helper cell mechanisms. Despite the potential impact on the IFN-γ axis, patients with IRF4 haploinsufficiency appear to be selectively susceptible to WD without susceptibility to other intracellular pathogens that rely on intact IFN-γ/STAT1 signaling (e.g. mycobacteria, *Salmonella*, etc.), implying that other parallel and/or compensatory immune mechanisms may play a role. Additional work is needed to further delineate the cell-specific mechanistic role of IRF4 in host defense against WD.

### EROS, NADPH oxidase, and innate immunity

The generation of reactive oxygen species (ROS) via the nicotinamide adenine dinucleotide (NADPH) complex is an essential component of innate host defense. Mutations in four of the genes encoding the protein components of the NADPH complex (gp91 ^phox^, p22 ^phox, ^p67 ^phox^, and p47 ^phox^) cause chronic granulomatous disease (CGD) manifested by an impaired phagocyte respiratory burst resulting in infectious susceptibility to bacteria and fungi ^31, 32^. Instead, inherited defects affecting the fifth protein, p40 ^phox^, result in severe inflammatory complications with modest infection susceptibility ^33^. A newly described transmembrane protein, EROS, has also been identified to play a key role in ROS production through stabilization of the gp91 ^phox^ and p22 ^phox^ heterodimer, cytochrome 588b ^34^ ( Figure 1). A cohort of eight individuals with a homozygous null mutation in *CYBC1* encoding EROS demonstrated a CGD-like disease with severe infections and associated colitis ^35^. Patients demonstrated absent cytochrome 588b surface expression and severely reduced neutrophil oxidative burst. Interestingly, Thomas *et al*. ^36^ reported a Saudi Arabian boy with biallelic mutations affecting *CYBC1* who, unlike the previously reported patients, suffered from recurrent sinopulmonary infections, autoimmune hemolytic anemia, and hemophagocytic lymphohistiocytosis (HLH) ( Table 1). Evaluation of the patient’s PBMCs showed absent EROS protein, while neutrophils demonstrated defective respiratory burst following stimulation with either phorbol 12-myristate 13-acetate (PMA) or zymosan. These cases provide additional evidence for EROS deficiency manifesting as a CGD-like disease but, given the heterogeneous clinical phenotype, also raise questions for additional roles that EROS may play in infection susceptibility and inflammation.

### Dominant activating RAC2 mutations and bacterial lung disease

RAC2 is a guanine nucleotide-binding protein belonging to the rho guanosine triphosphatase (GTPase) family. It is expressed in the hematopoietic compartment, where it promotes cell migration, adhesion, and oxygen radical production ^38, 39^ ( Figure 1). Human *RAC2* LOF mutations have been described to result in neutrophil defects ^40^, severe combined immunodeficiency ^41^, and a common variable immunodeficiency (CVID) clinical phenotype ^42^. Recently, *RAC2* dominant activating mutations were discovered in which patients suffered from recurrent bacterial sinopulmonary infections and bronchiectasis ^43, 44^. Patients exhibited lymphopenia possibly as a result of increased apoptosis ^43^ while neutrophils demonstrated excessive superoxide production ( Table 1), aberrant n-formyl methionyl-leucyl-phenylalanine (fMLF)-mediated chemotaxis, impaired macropinocytosis, and compromised actin remodeling *in vitro* ^44^ *.* This combined immunodeficiency underscores the importance of both neutrophil and lymphocyte function in the protection against bacterial lung disease.

### Teasing apart cellular signaling in the host response to mycobacteria

IFN-γ is critical for the control of intracellular pathogens including mycobacteria, *Salmonella*, *Listeria,* endemic fungi, and others ^45, 46^. Disorders of mendelian susceptibility to mycobacterial disease (MSMD) comprise 11 different genes to date, resulting in 21 different genetic diseases ^47^. Of these rare inherited disorders, autosomal-recessive (AR) IL-12 receptor β1 (IL-12Rβ1) deficiency accounts for the majority of cases ^48^. The *IL12RB1* gene encodes for IL-12Rβ1, which mediates IL-12 and IL-23 signaling via dimerization with IL-12Rβ2 and IL-23R components to form IL-12 and IL-23 receptors, respectively ^49, 50^ ( Figure 1). Similarly, mutations in *IL12B* encoding the IL-12 p40 subunit common to both IL-12 and IL-23 exhibit a clinical phenocopy of IL-12Rβ1 deficiency ^51^. Unlike most other MSMDs, a subset of patients with either of these two PIDs also carry susceptibility to chronic mucocutaneous candidiasis (CMC), thought to originate from compromised signaling via the IL-23/IL-17 pathway that is important for T _H_17 cell development and mucosal immunity ^52^. As both IL-12 and IL-23 signaling are impaired by these disorders, the individual contribution of each of these cytokines in the immune response to mycobacteria and mucosal candidiasis had remained unclear.

Through the identification of AR LOF mutations in either *IL12RB2* or *IL23R* affecting separate kindreds with MSMD without CMC, Martínez-Barricarte *et al*. underscored the importance of both IL-12 and IL-23 signaling pathways and their effects on IFN-γ mediated immunity ^53^ ( Table 1). Compared to healthy controls, patients deficient in IL-12Rβ2 demonstrated fewer memory T _H_1 cells and impaired T _H_1 differentiation *in vitro*, while IL-23R-deficient patient T cells showed impaired T _H_17 differentiation *in vitro*. The authors further investigated the impact of IL-12 and IL-23 on IFN-γ cellular responses by isolating several lymphoid cell types (i.e. B, CD4 ^+^ T, CD8 ^+^ T, γδ ^+^ T, and natural killer [NK] cells, type-1 innate lymphocyte cells [ILC1s], type-2 ILCs [ILC2s], type-3 ILCs [ILC3s], NKT cells, and mucosal-associated invariant T [MAIT] cells) from healthy donors. In response to IL-12 stimulation, B, T, γδ ^+^, and NK cells and ILC1s and ILC2s produced IFN-γ, whereas only NKT and MAIT cells preferentially produced IFN-γ in response to IL-23. Interestingly, ILC3s produced IFN-γ upon either IL-12 or IL-23 stimulation. Taken together, these recent findings demonstrate the cooperative effect of these cytokines among a panoply of cell types to promote IFN-γ-mediated protection from mycobacterial infection.

Like IL-12Rβ1 deficiency, AR TYK2 deficiency demonstrates impaired IFN-γ-mediated immunity through defective IL-12 and IL-23 signaling, resulting in intracellular bacterial (and/or viral) infections ^54, 55^ ( Figure 1). Recently, a common *TYK2* mutation, P1104A, with a homozygous allele frequency of ~1/600 Europeans, has been identified ^56^ ( Table 1). Unlike complete TYK2 deficiency, TYK2 P1104A exclusively impairs IL-23 responses due to compromised catalytic activity, thereby disrupting IFN-γ production and conferring risk of mycobacterial infection ^56^. In individuals of European ancestry, TYK2 P1104A may underlie a genetic basis for ~1% of tuberculosis cases; these findings indicate that inherited risk alleles may underpin infection susceptibility at the population level ^57^.

### RORC in the control of mycobacteria and Candida

RORC encodes the transcription factors RORγ and RORγt. RORγ is ubiquitously expressed, whereas the RORγt isoform is restricted to lymphocytes where it regulates IL-17-related gene transcription ( Figure 1) and T _H_17 T cell development ^58, 59^. Recently, biallelic mutations in *RORC* affecting seven individuals from three unrelated kindreds with CMC and atypical mycobacterial infections have been described ^60^. Patient cells demonstrated impaired IL-17A, IL-17F, and IL-22 responses, likely accounting for mucosal susceptibility to *Candida albicans.* In the evaluation of the etiology of mycobacterial vulnerability, PBMCs exposed to BCG and IL-12 demonstrated reduced IFN-γ secretion as a result of selectively impaired γδ and CCR6 ^+^CXCR3 ^+^CD4+ αβ T cell subsets. These findings show that *RORC* plays an integral role in both IL-17 and type II IFN pathways.

### JAK1: a complex immunodeficiency

The JAK–STAT pathway is central to many signaling cascades. Of the four JAKs, JAK1 is involved in multiple signaling pathways including IL-2, IL-4, IL-7, IL-9, IL-15, IL-21, and IL-27 as well as the IL-6 and IL-10 families of cytokines ^61^. As a result of JAK1’s widespread function, its deletion has been shown to be lethal in mice ^62^. To date, only a single patient with partial JAK1 deficiency has been reported ( Table 1), resulting in a combined immunodeficiency complicated by atypical mycobacterial osteomyelitis, sinopulmonary and skin infections, flat warts, and severe crusted scabies ^63^. Beyond infectious complications, the patient demonstrated developmental delay and an early death as a result of metastatic urothelial cancer at age 23. Functional studies demonstrated impaired JAK–STAT phosphorylation leading to compromised responses to multiple cytokines including IL-2 and type I and type II IFNs. These broad signaling defects are likely responsible for the T cell lymphopenia and viral and mycobacterial susceptibility, respectively.

## Recent insights gained from primary immunodeficiency disorders manifesting with viral infection susceptibility

### Novel primary immunodeficiency disorders of defective type I and III interferon signaling

Patients with germline defects in antiviral immunity have been shown to suffer from disseminated infection when exposed to live viral vaccine strains ^64^. Deficiencies in STAT1 ^65^, STAT2 ^66, 67^, or IFNAR2 ^68^ have been reported to predispose to severe infection from the live-attenuated measles, mumps, and rubella (MMR) vaccination strain. Shared among these disorders are important elements in the host antiviral response as mediated through type I (and type III) IFNs. Type I IFN signaling occurs via binding of the IFNAR, composed of two subunits, IFNAR1 and IFNAR2. IFNAR then activates JAK1 and TYK2, which phosphorylate STAT1 and STAT2. The resulting STAT1/STAT2 heterodimers translocate to the nucleus, where they assemble with IRF9 to form the IFN-stimulated gene factor 3 (ISGF3) complex, leading to the downstream transcription of IFN-stimulated genes (ISGs) responsible for antiviral host defense ^69^.

In a recent report, Hernandez *et al*. describe two patients with AR splice defects in *IFNAR1* who experienced severe infectious complications following vaccination with MMR and yellow fever vaccine strains, respectively ^70^. Patient cells demonstrated lack of IFNAR1 expression, unresponsiveness to type I IFN stimulation ( Table 1), and inability to control viral replication of measles and yellow fever viruses *in vitro*.

Novel advances in the understanding of IFN signaling have also been illuminated by the study of three patients with AR LOF *IRF9* mutations. A 5-year-old girl manifested severe influenza A virus (IAV) pneumonitis ^71^, and a 10-year-old boy suffered from recurrent viral infections resulting in neurological impairment and bronchiectasis. A third patient, the 6-month-old sister of the 10-year-old boy, was healthy at the time of the report, potentially suggestive of incomplete penetrance ^72^ ( Table 1). Patient cells demonstrated normal STAT1 and STAT2 phosphorylation but were unable to form the necessary ISGF3 complex, resulting in impaired induction of ISGs and failure to control viral replication *in vitro*.

Additional genetic evidence of influenza virus susceptibility comes from the recent description of three children with autosomal-dominant (AD) Toll-like receptor-3 (TLR3) deficiency complicated by IAV-induced acute respiratory distress syndrome ^73^. TLR3 acts as an intracellular sensor of double-stranded DNA (dsDNA), and its activation stimulates type I and III IFN production ^74^ ( Table 1). Dominant-negative *TLR3* mutations were initially recognized to cause defective intrinsic immunity of the central nervous system (CNS), impairing host IFN response to herpes simplex virus-1 (HSV-1) and underlying HSV encephalitis (HSE) in otherwise healthy children ^75^. As previously described for dominant-negative TLR3 deficiency ^75^, leukocytes from the patients with AD TLR3 deficiency demonstrated normal type I/III IFN activation. However, like patients with IRF9 ^71^ or IRF7 deficiency ^76^, fibroblasts and induced pluripotent stem cell-derived pulmonary epithelial cells (iPSC-PECs) failed to control IAV replication. Importantly, the phenotype could be rescued in iPSC-PECs when treated with IFN-α or IFN-λ1, highlighting the importance of intrinsic type I and III IFN immunity for viral control in the lung ^73^.

In addition to influenza, enteroviruses such as human rhinovirus (HRV) are major causes of respiratory viral infections, particularly in young children and immunocompromised adults ^77^. In the induction of protection against these viruses, MDA5 senses long fragments of cytosolic dsDNA, leading to the production of type I IFNs and pro-inflammatory IL-1 family of cytokines ^78, 79^. Several LOF mutations in *IFH1*, encoding MDA5, have been reported in patients suffering from severe respiratory viral infections ^80– 82^ ( Table 1). These mutations impaired downstream IFN signaling and led to defective control of HRV replication *in vitro* ^80, 81^.

Taken together, these data underscore the complex interplay found in host defense against a variety of viral pathogens and the crucial role that type I and III IFN signaling plays in the antiviral response.

### Novel defects of hyperactive immunity leading to viral infection-related morbidity

Recurrent respiratory papillomatosis (RRP) is a rare disease resulting in recurrent benign papillomas of the respiratory mucosa in otherwise healthy children and is typically caused by chronic infection with human papilloma virus (HPV) serotypes 6 or 11 ^83^. Thus far, an immunologic etiology for this disease has remained unknown. Drutman *et al*. reported two brothers with juvenile-onset RRP who harbor a private homozygous *NLRP1* mutation resulting in a GOF as demonstrated by increased IL-1β secretion and spontaneous inflammasome activation ^84^ ( Table 1). Interestingly, HPV6 or HPV11 was not detected in either patient, presumably because of an exaggerated antiviral response from inflammasome activation and inflammatory cytokine secretion. The cause of papilloma formation remains to be elucidated but may be secondary to the overproduction of inflammatory IL-1 family cytokines resulting in increased keratinocyte growth factor expression-mediated proliferation and papilloma formation ^85, 86^.

Less than one percent of children with hepatitis A viral (HAV) infection develop acute liver failure (ALF), and although liver transplantation has significantly improved outcomes, mortality remains greater than 10% ^87– 89^. HAV replication alone does not result in hepatocellular injury; instead, non-HAV-specific “bystander-activated” CD8 ^+^ T cells have been implicated in hepatocyte cytotoxicity ^90^. The mechanisms remain unclear, but evidence suggests that co-stimulation by type I IFNs and IL-18 results in potent T cell activation and IFN-γ secretion ^91, 92^. IL-18BP is important in the negative regulation of IFN-γ-mediated immune responses by binding IL-18 and preventing interaction with the IL-18 receptor. Belkaya *et al*. reported a child who succumbed to HAV-induced ALF as a result of AR IL-18BP deficiency, demonstrating that the mutant IL-18BP failed to block IL-18 activity ^93^ ( Table 1). Additionally, they provided important *in vitro* evidence that IL-18 mediates hepatotoxic effects via NK cell activation and cytotoxicity and that treatment with IL-18BP rescues the phenotype, indicating that recombinant IL-18BP may have therapeutic value in patients with ALF ^93^.

These reports emphasize the concept that unrestrained immune activation following viral infection may lead to serious clinical ramifications in patients with defects in immunoregulation or persistent inflammatory mediator production.

## Recent insights gained from PIDs manifesting with fungal infection susceptibility

### The mechanism of CARD9-dependent protection against central nervous system fungal invasion

CARD9 deficiency has been known to result in fungal-specific infection susceptibility with a predilection for mucocutaneous tissues and the CNS ^94, 95^. The CNS-targeted susceptibility to *Candida* infection was recently elucidated. Specifically, CARD9 deficiency results in impaired recruitment of neutrophils into the *Candida*-infected CNS in mice and humans. The resultant CNS neutropenia, which is detrimental for control of fungal CNS invasion, is not due to peripheral neutropenia or impaired neutrophil-intrinsic chemotaxis but instead is caused by defective induction of critical pro-inflammatory molecules in the fungal-infected CNS that abrogate neutrophil trafficking from the blood into the CNS ^96^. A recent follow-up study by Drummond *et al*. shed further light onto the cellular and molecular basis of the CNS neutrophil trafficking defect of CARD9 deficiency ^97^. Specifically, it was shown that, in response to the fungal peptide toxin Candidalysin, CARD9 is critical in CNS-resident microglia for the production of IL-1β via both transcriptional regulation and inflammasome activation, which in turn activates the production of neutrophil-recruiting CXCL1 by CNS-resident microglia and astrocytes to promote fungal control in the CNS. Taken together, two-step tissue-specific “hits” are operational in CARD9 deficiency; impaired IL-17 responses at the mucosal level allow for *Candida* translocation into the blood and from there to the CNS, where impaired IL-1β/CXCL1-dependent crosstalk between resident microglia and recruited neutrophils results in CNS fungal persistence.

### Fungal mucosal immunity and connective tissue disorders

CMC is manifested by superficial infections of the skin, nails, and mucous membranes caused by *Candida* species. As IL-17 is essential for antifungal mucosal immunity, inherited disorders underlying vulnerability to CMC are associated with impaired IL-17 signaling. Indeed, mutations in genes of the IL-17 signaling pathway, namely *IL-17RA* ^98^, *IL-17RC* ^99^, *IL-17F* ^98^, and *TRAF3IP2* ^100^, have been described in humans. Patients with AR *AIRE* deficiency resulting in autoimmune polyendocrinopathy-candidiasis-ectodermal dystrophy (APECED) ^101, 102^ carry neutralizing autoantibodies against IL-17F and IL-22 ^103, 104^. Other genetic disorders that lead to impaired T _H_17 development include dominant-negative *STAT3* mutations underlying Job’s syndrome ^105^, DOCK8 deficiency ^106^, the recently described deficiency in ZNF341 ( Table 1), a transcription factor that binds to the STAT3 promoter and results in defective STAT3 transcription and activity ^107, 108^, RORC deficiency ^60^, and *STAT1* GOF ^109, 110^.

In a recent report, Li *et al*. described a three-generational family with AD CMC and an atypical connective tissue disorder (CTD) due to mitogen-activated protein kinase-8 (MAPK8) haploinsufficiency ^111^. *MAPK8* encodes JNK1, a central component in the MAPK signaling pathway important in the transduction of cellular responses within many contexts ^112^, including transforming growth factor beta (TGF-β) signaling ^113^ and the IL-17A response pathway downstream of ACT1 ^100^. Evaluation of patient cells demonstrated reduced activation following IL-17A/F cytokine stimulation, impaired T _H_17 differentiation, and reduced proportion of T _H_17 cells *in vitro* ^111^. Moreover, patient fibroblasts showed impaired JNK1-mediated phosphorylation of activator protein-1 (AP-1) following TGF-β stimulation. These findings suggest that JNK1 haploinsufficiency results in combined impairment of IL-17 and TGF-β cellular responses, resulting in CMC and CTD.

## Conclusion

Research of PIDs provides an exciting opportunity to advance our understanding of human health. As technology continues to rapidly advance, so too does our sophistication in detecting subtle and once-unrecognized defects in the human immune response. As illustrated in the reviewed works above, certain defects may lend to a narrow range of infectious susceptibly often as a result of redundant host defense mechanisms selected throughout our evolution. Furthermore, with the increased access and availability to molecular sequencing platforms, the number of novel and interesting variants is expected to increase at an exponential rate. Evaluating these coding, non-coding, and epigenetic variants will further our understanding of the function and regulation of the human immune system. Deciphering the intricate interplay of hematopoietic and non-hematopoietic cells, organ-specific immunity, and environmental factors that influence infection susceptibility should help inform improved therapeutic and vaccination strategies in vulnerable patients.
